# Visual bodily signals as core devices for coordinating minds in interaction

**DOI:** 10.1098/rstb.2021.0094

**Published:** 2022-09-12

**Authors:** Judith Holler

**Affiliations:** ^1^ Max-Planck-Institut für Psycholinguistik, Nijmegen, The Netherlands; ^2^ Donders Centre for Brain, Cognition and Behaviour, Radboud University, Nijmegen, The Netherlands

**Keywords:** visual bodily signals, gesture, coordination, pragmatics, language evolution, precursors

## Abstract

The view put forward here is that visual bodily signals play a core role in human communication and the coordination of minds. Critically, this role goes far beyond referential and propositional meaning. The human communication system that we consider to be the explanandum in the evolution of language thus is not spoken language. It is, instead, a deeply multimodal, multilayered, multifunctional system that developed—and survived—owing to the extraordinary flexibility and adaptability that it endows us with. Beyond their undisputed iconic power, visual bodily signals (manual and head gestures, facial expressions, gaze, torso movements) fundamentally contribute to key pragmatic processes in modern human communication. This contribution becomes particularly evident with a focus that includes non-iconic manual signals, non-manual signals and signal combinations. Such a focus also needs to consider meaning encoded not just via iconic mappings, since kinematic modulations and interaction-bound meaning are additional properties equipping the body with striking pragmatic capacities. Some of these capacities, or its precursors, may have already been present in the last common ancestor we share with the great apes and may qualify as early versions of the components constituting the hypothesized interaction engine.

This article is part of the theme issue ‘Revisiting the human ‘interaction engine’: comparative approaches to social action coordination’.

## 1. The richness of utterance: beyond iconicity, towards multimodal pragmatics

When wondering about the role of gesture in the evolution of human language, opinions have been plentiful and diverse. Some consider its role as negligible if relevant at all (e.g. [1,2]). Scholars who ascribe a central role of gestures to how we have acquired language fall, broadly speaking, into two camps: those arguing for ‘gesture-supplanted-by-speech’ scenarios of language evolution, where gesture fulfilled a bridging function and its relevance largely withered away once a fully fledged linguistic system had been acquired, eventually leading to the speaking species that we are (e.g. [3–8]); and those who argue for language having evolved as a multimodal system, with the vocal and gestural modalities intertwined from the very beginning, and playing an integral role in communication also in modern human language (e.g. [9–19]). According to the former, spoken language is the evolutionary end product and the ultimate explanandum, while the latter considers this to be the unified speech-gesture system.

My aim here is to further corroborate this latter view by highlighting the substantial contribution visual bodily signals make to human pragmatic communication. Much of the focus to date has been on gestural iconicity and the encoding of semantic information as part of communicating referential meaning or propositional content when discussing the integral role of gesture in human language, *de novo* language creation and evolution (e.g. [12,13,20–37]). This may have led us to significantly underestimate the role of the body in the process of laying the foundations for the emergence of human language. Conveying propositional information, arguably, is one of the core building blocks of human communication. Studies focusing on iconicity (and deixis) in the emergence of language thus have revealed fundamental insights into the nature of human language, the evolution of symbolic communication, its learnability, conventionalization, systematization and how we transmit thoughts that go beyond the here and now. My point is certainly not that such a focus is to be argued with—quite the opposite; work on the role of gestural iconicity in language evolution and emergence has undisputable merit. However, the strong emphasis on iconicity, and consequentially on semantics, have distracted us from devoting attention to the role of visual bodily signals in pragmatic communication. Broadening our focus of enquiry will help us to recognize the full scope of the body's involvement in human communication.

This entails considering its contribution to a host of key ingredients required to make communication successful. Such an approach will facilitate connecting behavioural observations with theoretical frameworks recently put forward, such as the Interaction Engine Hypothesis [38,39]. The notion of the interaction engine refers to a set of behavioural and cognitive predispositions that constitute the roots of the advanced social interaction capabilities of the human species. These include signalling that an act is meant to communicate and that it is recognized as such, and that it can be deciphered for what it is supposed to mean—not just in terms of the semantic ‘bits’ of information encoded but in terms of what the speaker intends to achieve with communicating them at a particular moment in time (i.e. the pragmatic meaning and illocutionary force needs to be derived). An essential prerequisite for this is that speakers tailor messages to their specific addressees' requirements. Equally important are mechanisms that allow addressees to signal that they have understood or have trouble understanding, as well as the interactional structures that facilitate these processes, such as socially contingent actions and a turn-taking system. Section 2, therefore, reviews evidence which demonstrates the contribution of visual bodily signals to the following core pragmatic processes:

(i) signalling the intent to communicate;
(ii) communicating specific intentions and pragmatic meanings;
(iii) tailoring information to an interlocutor's knowledge (recipient design, common ground);
(iv) signalling understanding (grounding);
(v) signalling and repairing trouble in understanding; and
(vi) calibrating communicative action to the interaction in progress, moment-by-moment (coordination of sequential actions, turn-taking).

To gain these insights, we require an approach that goes beyond a focus on the purely semantic information in the visual modality. This means: (i) focusing on the pragmatic functions of gestures even when these themselves are iconic or deictic in nature; (ii) considering manual gestures other than iconic and pointing gestures, such as those defined as pragmatic and interactive gestures; (iii) analysing visual signals carried by articulators other than the hands, especially the face, and the combination of signals into visual–visual or multimodal composites; (iv) measuring the contribution to communication not only based on depiction and deixis (the what), but also based on analyses of movement kinematics and structural aspects of signal form (the how); and (v) applying a socially contextualized perspective able to capture the detailed interactional and reciprocal embeddings of visual bodily signals.

Such an approach may also offer new avenues for cross-species comparisons (§3). As noted by Fröhlich *et al.* [9], many of the gestures typically theorized about have rather little in common with those used by non-human primates. An interactionally-grounded approach that puts the pragmatics of multimodal behaviour centre-stage may thus offer new avenues for advancing our understanding of how we coordinate minds in human communication and how this ability may have evolved (§4).

## 2. Visual bodily signals as coordination devices in human communication

This section aims to illustrate how broadening our focus in the manner described above facilitates appreciating the contribution of visual bodily signals to core components of human communication—that is, their function as coordination devices in joint actions [19]. For each of the six components listed in the previous section (here constituting §§2a–2f), some evidence for the contribution of visual bodily signals is given, ranging from manual gestures, torso movements, head gestures and eye gaze, to facial signals, as well as composites (i.e. signal combinations) involving more than one type of articulator or method of signalling [19].

One disclaimer is necessary at this juncture: what follows is a non-exhaustive summary of some of the ways in which the body forms part of a selection of processes key to human communication. In its brevity, it does not do justice to the full breadth of relevant extant research, and there will certainly be omissions owing to the author's own shortcomings. Nor does it do justice to the rich insights that can be gained from microscopic, detailed and holistic analyses of situated human interaction yielded by conversation analytical techniques, since much more in-depth treatments are necessary to reveal them. Nevertheless, I hope it will become clear from this brief review how manifold the functions of visual bodily gestures are and how vital their contribution is to the pragmatic processes that they form an inherent part of.^1^

### (a) Visually signalling the intent to communicate

To facilitate coordination, interlocutors produce behaviours in such a way that allows them to be identified as being intended to communicate, thus contrasting them with behaviours that are not.

#### (i) Manual gestures

In [40], participants performed manual gestures and actions either for an addressee to learn from them or in a more self-serving context. Movements in the more communicative context were larger and more complex in structure than less communicative movements. Pointing gestures, too, differ kinematically depending on whether they are more or less communicatively intended, with communicative pointing gestures being produced more slowly (duration from onset to the maximum point of extension) and with less velocity than less communicative pointing gestures, and being held longer in their pointing position [41]. Since these studies manipulated the extent to which the signals were relevant for addressees, there is reason to believe that kinematic variation of this kind is produced by speakers with the intent that their interlocutor perceives the behaviour as ostensive and thus as communicatively relevant and intended [42].

#### (ii) Torso movements

Looking beyond the hands, body orientation is an important give-away as to the communicative intent of another agent. The body being oriented towards someone signals address, the intent to initiate and maintain interaction [43–47].

#### (iii) Gaze

Person-directed gaze is another signal conveying the intent to communicate or initiate interaction [40,48–53] (and person-directed face orientation can have a similar function).

#### (iv) Composites

Gaze direction also interacts with the directional positioning of other bodyparts (torso, legs, head) resulting in complex gestalts. These are core to signalling the intent to engage in or maintain interaction and the dynamic coordination of participation frameworks [43,54–58]. Speaker gaze directed towards co-speech gestures also highlights their communicative intendedness for the addressee [59–63].

Thus, one of the most primordial of processes—signalling the intent to communicate and initiating interaction—is done bodily in various ways, using a range of different articulators, their orientation, and kinematic variations.

### (b) Communicating pragmatic meaning and illocutionary force with visual bodily signals

The ultimate prerequisite for communication to be successful is for an interlocutor to recognize the message as what the speaker intends it to mean [64,65]. A look at conversation, the natural home of human language use, highlights the pragmatic meaning contribution of visual bodily signals.

#### (i) Manual gestures

**Figure 1.**
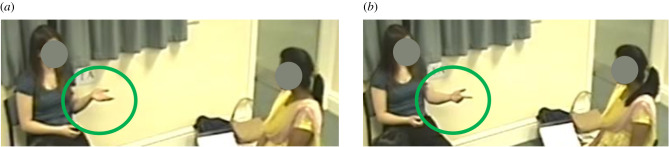
Example of an OHS (open hand supine, or palm-up-open-hand) gesture (*a*), and example of a pointing gesture directed at the addressee as a way of marking information constituting mutually shared knowledge which the speaker is referring to verbally (common ground pointing gesture (CG-point)) (*b*).

Pointing gestures can communicate indirect requests both in the presence and in the absence of speech [66,67]. Manual gestures also contribute to communicating illocutionary force [65] through palm orientation. Kendon [68] proposed the notion of ‘gesture families’, i.e. groupings of gestures which share kinesic features (such as hand shape, orientation and movement pattern) and are related in meaning and pragmatic function. One example is the ‘open hand supine’ (OHS) family consisting of gestures performed with an open, flat hand and the arm in the supine position (also ‘palm-up-open-hand gestures' [69]) (figure 1). OHS gestures are associated with the themes of giving, offering, showing/presenting, requesting or receiving—i.e. the metaphorical embodiment of a non-present entity being placed on the flat palm (see also [70–72]). OHS gestures may be directed towards the space in front of the speaker (e.g. to offer an idea or explanation), towards the interlocutor (e.g. to request a response) or to entities or locations in the environment (e.g. to present or display a person or object). Another group of manual gestures is characterized by the palm facing down or forwards, typically combined with lateral motion or motion away from the body, clearing the body space from unwanted entities in a metaphorical way [68,73,74]. Such gestures act as embodied forms of negation, declination, refusal and negative assessments.

These examples illustrate manual gestures contributing to the illocutionary force of utterances by clarifying if an utterance should be interpreted as an offer, a request, a declination and such like. Critically, the illocutionary force of such recurrent gesture forms often remains implicit when considering just the accompanying speech [75], thus highlighting gestures’ core pragmatic contribution to the conversation. Aside from illocutionary force, manual gestures contribute to pragmatics also by adding emphasis and contrast to elements in speech (e.g. beats [34]), something Kendon referred to as the ‘parsing’ function of gestures [68].

#### (ii) Torso movements

The body's contribution to communicating pragmatic meaning is just as evident when we look beyond manual gestures. Research in the domain of human sensorimotor communication and joint action has revealed that point-light displays of body movements (in the absence of speech) allow observers to decode information about specific intentions, such as ordering someone to do something, requesting or offering an object [76,77]. In conversational context, torso movements were found to differ between categories of social actions, with questions expressing a stance or sentiment being accompanied by larger torso movements than requests for information, for example. This may be related to the act of distancing oneself from or showing agreement with certain attitudes [78].

#### (iii) Head gestures

The fundamental pragmatic functions of affirming and negating are often accomplished through highly conventionalized movements of the head, such as nods and shakes, and addressees use head nods to signal agreement and to confirm receipt and understanding [79,80]. These may perhaps be considered the prototypical ones, but the rich repertoire of complex head movements in conversation has long been acknowledged, and the wide range of pragmatic functions they fulfil carefully documented (see e.g. [81]; Heylen [82,83]; Wagner *et al.* [84]).

#### (iv) Gaze

Gaze direction, too, plays a significant role. For example, gaze aversion in conversation can mark dispreferred responses (such as declining an offer) and may occur considerably earlier than the verbal response itself [85]. Also, rolling eyes can mark statements as ironic [86].

#### (v) Facial signals

One of the main bearers of pragmatic information during conversation is the face. For many decades, facial expressions were the focus of emotion research, but recent years have seen this starting to shift. Eyebrow movements are a frequent question marker in spoken and signed languages alike [87–89]. Frowns, too, are a common occurrence [90] and can foreshadow trouble in understanding [91] and form a contrast with eyebrow raises to mark different forms of clarification requests [92]. Squints also often mark questionhood [93,94]. Eyebrow raises, eyebrow flashes and expressive mouth movements have been associated with utterances to be interpreted as sarcasm and irony [86,95].

#### (vi) Composites

Pragmatic manual gestures often combine with other visual signals, such as manual negation gestures co-occurring with headshakes, and palm-up gestures with shoulder shrugs and facial signals [68]. The combination of signals acting as gestalts is particularly prevalent in the face. Squints, for example, frequently co-occur with eyebrow frowns in marking questionhood [90]. The ‘not-face’ consists of an interplay of several signals even, most commonly eyebrow frown and pushed up chin muscles, plus pulled back corners of the mouth or pressed lips, expressing negative moral judgement [96]. The ‘facial shrug’ involves one corner of the mouth being pulled back plus an eyebrow flash (but mouth and eyebrow components can also occur on their own), translating as ‘I don't know’, ‘oh well’ or expressions of dislike [97,98]. The ‘thinking face’ involves a blank, puzzled or concentrated face with furrowed brows or raised eyebrows combined with averted gaze [98,99], typically accompanying word searches.

In summary, a wide range of visual bodily signals contributes information that specifies the pragmatic meaning and illocutionary force of an utterance. Importantly, the information the body contributes is often not encoded in the verbal modality and frequently occurs before or at the utterance beginning, thus underlining their communicative import.

### (c) Producing recipient-designed utterances with visual bodily signals

The notion of recipient or audience design in communication refers to speakers' sensitivity to the co-participants in an interaction and to the adaptation in communicative behaviour this leads to [100,101]. Crucially, this adaption entails speakers designing their utterances such that they match the knowledge, beliefs and assumptions of the participants they want their message to reach. Designing utterances with the intended recipient(s) in mind is a fundamental pragmatic process, and successful communication depends on it [7,19]. There is plenty of evidence that speakers adapt their signalling in response to environmental factors (such as noise or visibility), but even more convincing is the versatile ability of manual gestures in crafting messages that are tailored to fit addressees’ informational needs.

#### (i) Manual gestures

A number of studies have shown that speakers draw on the gestural modality in a manner that takes into account common ground—that is, the knowledge, beliefs and assumptions that interlocutors mutually share [19]. Typically, this involves a reduction in gesture frequency (and sometimes gesture rate [102]) and information content [103–105], when common ground between interlocutors exists (but see [106]). Speakers also mark reference to shared knowledge through gesture form. Gestures are often smaller [104,107,108], less precise [103,108] or occur lower in gesture space [109] when referring to information that already is in the interlocutors' common ground (with some exceptions [102]). Similarly, speakers use gesture duration to contrast given with new information in referential communication (see [110], and references relating to other ways of gestures marking information status therein).

Further, some non-iconic, interactive palm-up gestures serve the purpose of marking what someone is saying as being a citation of something said earlier [111]. And interactive pointing gestures towards the addressee (figure 1) can mark information as references to common ground, often without explicit verbal reference to the information's common ground status [112].

#### (ii) Facial signals

Much less is known about facial signalling of common ground. In some signed languages, squints can mark information as common ground and its degree of accessibility [113]. Whether squints and other facial signals function similarly together with spoken language is a highly interesting question for future research.

In summary, the fundamental process of designing messages appropriate for specific recipients is a multimodal phenomenon. Much of this process rests on kinematic form changes in gesture being pragmatically instrumental, in addition to or *in lieu* of adaptions in speech, and it goes beyond the use of iconic manual gestures.

### (d) Grounding information with the body

Achieving mutual understanding in interaction is foundational to human communication. Core to this is the process of grounding, i.e. reaching the mutual belief that a conversational contribution has been understood by all participants [114]. One common way of grounding information is with minimal verbal responses, such as ‘mhm’ or ‘yeah’, signalling understanding and allowing a conversation to proceed [115]. Producing a next speaking turn that pragmatically fits the previous one, too, signals understanding, as does responding with a fitting instrumental action if warranted by the context [19,116,117].

#### (i) Manual gestures

Interlocutors use iconic depictions to signal understanding through alignment in gesture form ([118–122], see [18] for a theoretical account linking such behavioural with cognitive alignment). For example, a speaker may refer to a man holding a big grocery bag by depicting the action of holding such a large bag themselves. Their interlocutor may reuse that very gesture later on in the dialogue to refer to the same character. Such repeated gestures give the clear impression that interlocutors have mutually shared understanding of whom they are referring to. The gestures may even do so more unambiguously than verbal references, especially when words are not aligned, are not produced at all, or change in order to propose a different verbal conceptualization, where the gestures then provide the ‘visual anchor’ of alignment [118, p. 143]. Reusing an interlocutor's gestures may be a particularly efficient form of grounding, and it is deemed intentional, thus differentiating this process from other forms of behaviour matching such as foot tapping [118]. Note that repeated gestures are not about semantic information being conveyed or iconically mapped afresh—the reuse of the same gestural form is critical (with some leeway [118,119]) so that it is recognized as a reference to something said earlier by the other interlocutor. The gestures thus obtain their grounding function through repetition across interlocutors.

Manual gestures with interactive functions, typically performed as palm-up gestures, also play a role in grounding, in two different ways [111]. One function is seeking a response from the addressee that would indicate understanding (thus functioning similarly to the words ‘you know?’). The other relates to palm-up interactive gestures that occur in a different sequential position: Bavelas *et al.*’s [123] micro-analysis of the grounding process has exposed not just two steps (speaker's contribution and addressee's signal of understanding) but also a third, namely the speaker acknowledging the addressee's understanding, claimed to be a crucial step in establishing *mutually* shared understanding. Interactive palm-up gestures in this third-step-position thus paraphrase as ‘I see that you understood me’ [123, p. 397]. Critically, the information conveyed by the interactive grounding gestures is typically not verbalized.

#### (ii) Head gestures

Head gestures are incredibly frequent in conversation, especially as part of the addressee's signal repertoire [79,84,124]. Malisz *et al.* [79] found that the majority of listener head gestures were nods (often consisting of multiple nodding cycles), used for different pragmatic functions, such as displaying continued attention and confirming understanding. The form of the head gestures seemed to disambiguate between those two functions, with head movements consisting of an upward motion followed by a downward motion (i.e. reverse nods) being more specifically used for confirming understanding. Both of these types of head movements occurred in the second position, i.e. as the addressee's response to information conveyed by the speaker. Head gestures also play a prominent role in the third position of grounding sequences, i.e. as acknowledgements of the addressee signalling understanding, thus functioning similarly to the third position palm-up interactive gestures described above [123].

#### (iii) Facial signals

Smiles, too, can function as grounding signals [125] and frequently form part of the three-step grounding process (see [123] for examples). Eye blinks, too, can contribute to the process of grounding [126,127]. From what we know so far, blinks occur mainly in the second position, i.e. as addressees' responses to speakers’ utterances, especially during extended speaking turns [127]. Importantly, the subtle kinematics of eyeblinks appear to make a difference, with long blinks (duration greater than 410 ms) functioning more clearly as grounding signals than short blinks, at least in experimental settings [126]. Eyebrow flashes, too, can function as signals for grounding. In Yélî Dnye (a Papuan language isolate spoken on Rossel Island), eyebrow flashes are conventionalized, meaning ‘yes, continue’, or function as an affirmative response [128,129].

#### (iv) Composites

Noteworthy is the frequent co-occurrence of blinks with nods [126,127], or blinks with eyebrow flashes [129], which may lead to an amplified communicative effect. Other facial signals may easily combine into more powerful grounding devices, too, such as shoulder shrugging with a cute face or rolling eyes [123]. Addressee facial ‘displays’ can also serve the grounding process, such as wincing in response to someone describing a painful experience, or a facial expressions of concern or fear in response to elements of close call stories [130,131]. Such ‘specific listener responses' [131] signal understanding of the message (as well as empathy), and possibly more unambiguously so than ‘generic listener responses’ (e.g. ‘mhm’, nods).

While gaze may not function as a grounding signal in itself, the significance of visual signals for grounding is underlined by the fact that speakers actively elicit them by looking at the addressee, thus creating brief time windows of mutual gaze during which the addressee responses occur [131]. Thus, manual gestures, head movements and facial signals play an important role in the process of grounding, a *sine qua non* in conversational interaction [19].

### (e) Visual bodily signals for initiating repair and responding to repair initiations

Despite being able to draw upon mechanisms for grounding that are beneficial for achieving mutual understanding in conversation, troubles in understanding are common [132]. Causes include trouble in hearing and in grasping the actual content of an utterance. Part of the process of achieving mutual understanding is repair mechanisms for such instances in which communication goes awry [133]. Vocally, interlocutors can refashion their own utterances to undertake repairs (self-repair), or issue requests for repair by asking ‘huh’, ‘what?’, ‘who?’ and so forth (other-initiated repair).

#### (i) Manual gestures

In face-to-face interaction, visual signals form an integral part of the repair strategies interlocutors have at their disposal [134]. For example, speakers' rate of self-repair and time spent manually gesturing is positively correlated [135], and when encountering dysfluencies associated with miscommunications, speakers produce manual gestures higher up in gesture space [136], presumably to foreground the gestural information in resolving the communicative trouble. When attempting to self-repair, speakers also use gestures to elicit helping responses from their addressees [111,137,138], usually in the form of interactive gestures expressing that the speaker is in some kind of communicative trouble (e.g. palm-up gestures or rubbing index finger and thumb together, held up in interactive space to invite a word suggestion).

**Figure 2.**
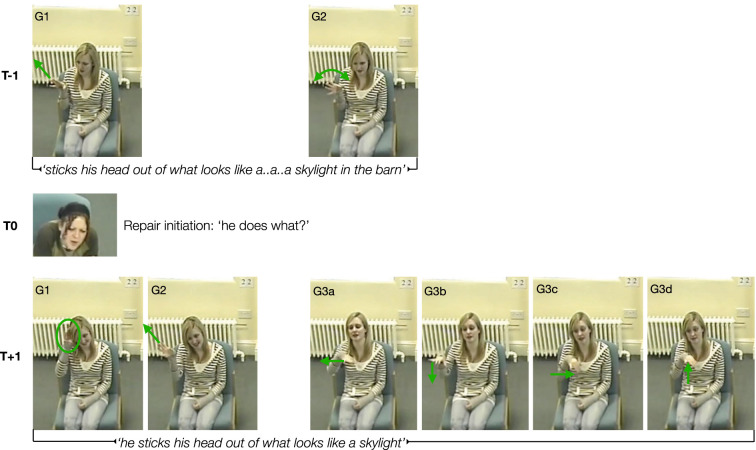
Illustration of a multimodal repair sequence. T − 1, trouble source; T0, repair initiation; T + 1, repair solution [139]; G, gesture (G3a − G3d = gestural subcomponents). The speaker says (T − 1) ‘[*he goes up another level and*] *sticks his head out of what looks like a a a skylight in the barn*’ to describe a little boy who climbed up to the top of a barn looking out of a window in the roof. In response to the addressee initiating repair (T0) by asking *‘he does what?*’, the original speaker says (T + 1) ‘*he sticks his head out of what looks like a skylight’.* Note that the speech at T − 1 and T + 1 differs marginally, the change consisting mainly of the last three words (in the barn) having been dropped and the utterance not being dysfluent. The accompanying gestures, however, do most of the repair work. The utterance at T − 1 includes two gestures. The first involves the flat hand, palm down, moving diagonally upwards and forwards depicting the boy's head moving up through the roof window. The second gesture is a pragmatic one and involves the hand, palm facing down and fingers spread, turning in the wrist (from left to right, 3×) which seems to convey uncertainty about whether the term ‘skylight’ is the best fit. Both gestures get revised as part of the utterance in T + 1: the first gesture now involves the hand, held in the same way as before, moving first to the speaker's own head before then moving upwards and forwards. This added movement clarifies that the upward moving hand is meant to depict a head moving up. The second gesture changes from a pragmatic to an iconic one, now outlining the square shape of the opening in the roof, presumably to illustrate its window-like features.

When responding to repair initiations, the manual modality also plays a role. While speakers may not necessarily gesture more in response to clarification requests [61,138], they do adjust their manual gestures to address the recipient's informational requirements. In the example above (figure 2) the speaker responds multimodally to a repair initiation from the addressee. Verbally, the speaker actually more or less repeats what was said (i.e. the trouble source). The burden of the repair work is accomplished gesturally by adjusting the form, and consequently the meaning, of the two gestures that formed part of the original utterance. In addition to adaptations in the semantic information encoded, manual gestures speakers use to respond to clarification requests can also differ in their kinematic realization; typically they then become more precise, larger or are performed in more visible areas of gesture space [61]. Only a consideration of the situated, pragmatic use and function of the gestures and the change in gestural form reveals such contributions to the repair process.

Addressees also make substantial use of manual gestures when *initiating* the repair. Healey *et al*. [138] showed that addressees' hand movements not only increased and became faster, but their behaviour also changed from more generic feedback (e.g. nods) to content specific manual gestures when issuing clarification requests. Also, Mortensen [140] described addressees using a specific type of iconic gesture, a cupped hand placed behind the ear, pragmatically in order to initiate repairs.

#### (ii) Torso movements

Movements of the torso, too, accompany repair initiations. Addressees may lean forward to intervene with questions, seek clarification, holding the lean until a response or clarification has been obtained [141,142]. Forward torso leans also occur when speakers attempt to provide repair solutions, especially as pursuits (i.e. when understanding in response to initial repair attempts is not displayed) [143].

#### (iii) Head movements

Speakers producing self-repairs nod more, and the primary and secondary (in multi-party conversation) addressee(s) also increase their nodding to help solve the trouble in utterance formulation [144]. Addressees also use head movements to *initiate* repair, including lateral turn or tilts and forward movements of the head to signal problems in understanding and to request clarification [142,145,146].

#### (iv) Facial signals

The face plays a prevalent role in addressees’ repair initiations also, particularly eyebrows [92,146]. There even appears to be a fine-grained association between eyebrow raises and open repair initiations (e.g. ‘huh?’, ‘what?’) and eyebrow frowns with restricted repair initiations (e.g. ‘he did what?’), thus hinting at intricate connections between the precise form of eyebrow movement and the type of response desired [92]. In some cultures, eyebrow flashes are used to confirm a speaker's successful repair [129].

#### (v) Composites

Eyebrow movements can form part of more complex facial displays for repair initiations, such as raised eyebrows combined with averted gaze and corners of the mouth pulled down [146].

In addition to the presence of the various bodily movements being associated with repair, their *absence*, too, can carry a signalling function. When addressees initiate repair, they may temporarily pause any bodily movement (including gaze, head, torso lean, eyebrows or manual movements) keeping them in a static hold position [147], which can result in something like the ‘freeze-look’ [148]. Interlocutors respond to the embodied absence of responses, for instance by expanding and refashioning their preceding turn to repair the communication problem [149].

The above shows that the body may be used in various ways to address problems in understanding, including addressees signalling trouble in understanding and speakers repairing troubles in speaking and understanding.

### (f) Coordinating sequential interaction with visual bodily signals

The vehicle for exchanging contributions in conversation is by taking turns at talking [101]. While producing speech in an alternating fashion may seem trivial at first sight, a closer look at what it entails reveals the complexities of the process. The gaps between consecutive turns are so short that the next speakers must start planning their contribution in parallel with the current turn that is still underway in order to launch it on time. This requires the prediction of upcoming content, as well as its approximate ending [150]. In face-to-face interaction, the body is very much involved in these processes.

#### (i) Manual gestures

Gestures conveying meaning are timed such that they significantly precede corresponding speech. This endows them with the power to project turn content, which may help early next turn planning (i.e. what to say) [151]. Further, manual gestures contribute to the alternation and timing of turns (i.e. when to speak). Duncan [152] showed that when the hands are actively involved in gesticulation they act as strong signals suppressing potential next speakers from taking the floor, while not gesturing yields the floor. In line with the latter, there is evidence that gesture retractions are associated with turn transitions rather than turn continuations [153], and retractions prior to turn end lead to shorter transitions than gestures retracted after turn completion [154]. Interestingly, certain gestures do not act as attempt-suppressing signals but in the opposite way, such as palm-up gestures offering the turn [68,71,111,155]. Moreover, participants in the conversation can gesturally signal their intention to speak next, such as by pointing to targets in a shared visual space while another speaker's turn is still underway [156].

#### (ii) Gaze

Some studies suggest that speakers look at addressees when they wish to hand over the turn, or that such behaviour solicits turn-taking attempts from the addressee [152,157]. In multi-party interactions, gaze direction can be an effective signal for the next speaker selection [51,158]. However, others have argued that gaze is organized at the level of sequences of action, such as gaze being directed at the addressee during the initiation of a question and averted only on completion of the response, rather than the end of the question turn [159,160]. Gaze thus seems to play a crucial role in the sequential coordination of talk in face-to-face interaction and may do so at various levels.

#### (iii) Composites

Visual bodily signals may also combine in intricate ways. Duncan proposed that the more floor yielding signals co-occur, the more likely are attempts from the addressee to take the floor [152], but that the floor-keeping signal of gesturing trumps the occurrence of any turn yielding signals. However, certain interactive gestures offering or handing over the turn must be an exception to this, and their effect may be enhanced when speakers gaze or bodily orient towards their addressee. Prosodic information and speech content are important cues in the verbal modality [150,161] and interact with visual signals, presumably leading to complex multimodal gestalts that govern turn-taking in face-to-face interaction.

## 3. The quest for similarities between human and non-human primate communication

There is clear evidence for some degree of iconic and deictic gestural behaviour in non-human primates [162–167], and such discoveries are highly interesting. However, looking for points of comparison based on human pragmatic and interactive signalling allows us to potentially see further in terms of the evolutionary precursors to human language. As in humans, visual signalling in non-human primates is not confined to the hands. A wide range of articulators form an integral part of the repertoire, including eyes/gaze, mouth, head, arms, torso, chest, back, feet, legs or the entire body at once (e.g. [168–172]). Recently, the face has also been increasingly studied in great apes. While facial signals have been assumed to be rather fixed and reflexive, accumulating evidence points towards considerable complexity, flexibility and intentionality [173–175]. The following paragraphs hypothetically suggest some parallels that arise when considering communication by means of non-iconic visual signals and kinematic variation. They also point to recent studies that have adopted a more fine-grained analytic approach focused on interactive, reciprocal processes to further our understanding of non-human primate communication. An approach that moves beyond iconicity and involving detailed analyses of visual signalling in interactive *situ* may shed more light on some interesting parallels and potential precursors of core components foundational to pragmatic communication in humans (§§3a–3g).

### (a) Visually signalling an intent to communicate

Similar to humans, body and face orientation as well as gaze direction can function as address and interaction-inviting signals in non-human primates [14,176–180]. Further similarities are observable when looking at movement kinematics. For example, gaze of prolonged duration can signal communicative desires in gorillas [14], and a study on gibbons showed that the duration of facial expressions was longer in social contexts and when they could be visually perceived by a conspecific [175]. The amplitude of movements can also be linked to communicative motivations, such as observed in the ‘directed scratch’, a kinematically exaggerated scratching action understood as a request for grooming which differs from the purely instrumental action of scratching oneself [181]. Modulations of kinematic features such as signal duration or movement amplitude in connection with social and communicative motivations closely resemble the kinematic modulation of human behaviour, as seen above [40,41,110,126,127].

### (b) Communicating pragmatic meaning and illocutionary force with visual bodily signals

One striking case of similarity in visual bodily signalling is the use of palm-up gestures in humans [68,69], orangutans [182] and chimpanzees [183] when making requests (e.g. food), one of the most fundamental of communicative actions. Deictic gestures, too, can be used by bonobos and chimpanzees to direct attention and make requests [162,163,184], as in humans [19,66,67].

The pragmatic functions of head gestures also bear some close resemblance to those of humans, such as bonobos preventing others' actions with head shakes, a presumed potential precursor to human visual signals of negation [185,186].

There is also evidence for the combination of movements into facial displays in non-human primates. The development of facial behaviour coding tools (e.g. ChimpFACS [187], OrangFACS [188] and GibbonFACS [189]) have propelled this research significantly forwards by allowing for fine-grained kinematic analyses [190]. This has yielded insights into configurational patterns of muscle activity forming facial expressions comparable to humans, at least in their morphological basis [187], as well as differences across species [188]. Composite signals can also be found beyond facial expressions. Oña *et al.* [191] investigated the combination of two different arm gestures with facial expressions in chimpanzees. While both gestures elicited affiliative responses when used on their own, the combination with a bared teeth facial expression enhanced this affiliative effect when paired with a stretched arm gesture, but eliminated the affiliative responses when combined with a bent arm gesture. Moreover, when embracing a multimodal view of non-human primate communication (e.g. [183,192–196]), evidence of cross-modal signal combinations can be found. Genty [194] found that contest hoot vocalisations were more frequently combined with gestures classed as ‘soft’ (e.g. touch) than ‘rough’ (e.g. kick) when used during friendly play, but the reverse applied to contest hoots used in agonistic contexts. Further, Eckert *et al*. [197] refer to reports on the sign-trained gorilla Koko, who used facial signals (the play-face) to contextualize what appeared to be intentionally produced wrong manual signs as acts of teasing.

Findings from these studies not only offer important new insights into the compositionality of non-human primates' communicative acts, a core feature of human language. They also show that compositional differences can map onto differences in pragmatic function, which is another striking resemblance (e.g. [90,93–95]).

### (c) Producing recipient-designed utterances with visual bodily signals

Great ape gestural communication boasts some of the basic ingredients for recipient design, such as awareness of the recipient's visual attentional state, as evidenced by apes moving around an experimenter so they can see their gestures [198] and by young chimpanzees' cross-modal gestural adjustments (silent-visual versus audible-or-contact gestures) for their visually attentive/inattentive mothers [199]. Expanding such analyses to further domains of pragmatic behaviour and cognition, therefore, seems worthwhile. For example, looking for kinematic modulations of gestural behaviour when communicating about information that is or is not in common ground (e.g. [200,201]) paralleling those found in humans (size, space, precision, as seen above) may shed new light on the extent to which great apes consider their recipients’ knowledge or state of mind. Interesting, too, would be analyses focused on incremental common ground (which builds up as a consequence of shared interaction history [19]). Recent findings show that bonobos are able to take into account this form of common ground, using more established (and successful) signals with interlocutors who share interaction history with them than with interlocutors who do not, while the reverse pattern was found for the use of novel signals [202]. An important question is whether such repeated signals bear any similarities to when and how humans repeat their gestures, and whether repetitions of the same gesture forms across interlocutors [118–122] occur in apes at all. If they do, one may wonder if such repetitions act in any way as a form of grounding (indicating communicative ‘success’ and mutual understanding) as they seem to do in human communication [118].

### (d) Visual bodily signals for grounding and repair

Further similarities between human and non-human primate communication can be gleaned from reciprocal behaviour in the light of communicative success or lack thereof. Response waiting followed by repetition or adjustment of gestural behaviour when communication was unsuccessful—behaviours observable in great apes [9,14,195,202–205]—resembles humans' close observation of their interlocutors for evidence of understanding and communicative success [116] and gestural adjustments as strategies for repair when needed (figure 2; [61]). A particularly interesting example is the observation of orangutans trying to address problems in understanding by adapting their signalling to the degree of (mis)understanding evident from their interlocutor's behaviour. Partial misunderstanding led to adaptations that involved narrowing down the range of signals used, focusing on a subset of signals previously employed, but complete misunderstanding was met with broadening the range of signals used and avoiding unsuccessful ones [205]. It is possible that the ability for such adaptations may be the evolutionary foundation on which the capacity for detailed semantic and pragmatic gestural adaptations observable in humans (e.g. figure 2) may have built. Future studies investigating the link between specific informational requirements for solving misunderstandings and non-human primates' detailed gestural adaptations (or lack thereof) to address these may shed more light on this issue.

While specific vocal repair initiations in great apes may not exist, changes in the dynamics of movement that hint at problems in uptake—including holds or tacit forward movements of head or torso, as seen in human communication [141,147]—are much more of a possibility. One interesting question is whether the observation that repair by non-human primates tends to be self- rather than other-initiated (Heesen *et al*. [206]) is confirmed if we apply a micro-analytic perspective including fine-grained movement kinematics. An interesting question, too, is whether it may be possible to find kinematic correlates of goal achievement in ape behaviour following responses to instances of persistence or elaboration, similar to the release of bodily articulators that were held in a static or forward position [147]. Equally interesting would be a focus on analogies (or differences) to the three-step grounding process (A–B–A, i.e. interactants acknowledging each other's communicative moves and ratifying them as satisfactory) evident in human interaction, as seen above [123].

### (e) Coordinating sequential interaction with visual bodily signals

One domain in which a sensitivity for sequential, reciprocal action has been observed throughout much of the animal kingdom is that of turn-taking, vocally and gesturally [207,208]. Similarly to humans, turn sequences in apes are frequently accompanied by gaze [180,208], an association also seen in collaboratively breeding marmoset monkeys (Burkart *et al*. [209]). One interesting focus for future cross-species comparisons is the micro-organization of articulators and signals in the sequential coordination of interaction, such as the precise temporal interplay of gesture and gaze (and perhaps vocalizations).

We already know from recent studies that gestural behaviour in non-human primate interactions is often characterized by a high degree of mutual calibration on a temporally fine-grained scale, creating the opportunity for moment-by-moment reciprocal feedback and adaptability, similar to the behavioural processes observable in human interaction. Recent work has suggested that, at least for chimpanzees, such processes may be much more characteristic than communication based on a fixed species-specific repertoire of gestures, especially in mother–infant interactions [164,210,211]. The Social Negotiation Hypothesis [210,211] is based on data suggestive of idiosyncratic and flexible, dyad-specific and context-specific gestural communication and proposes that ‘interactants mutually shape—or [*sic*.] ‘socially negotiate’—the outcome of each gestural interaction in real time’ [211, pp. 557–558]. For example, analyses of great ape interactions show that gestures may be adapted based on factors such as the attitudinal state of the infant (coordinative/discoordinative) resulting in different intensities of a push gesture [164], or the mother's consideration of whether the context requires the need for carrying the infant [210].

Detailed analyses of behaviour among non-human primates, also applying methods informed by conversation analysis [58,206,212,213] reveal their interactions to be orderly, reciprocal, micro-coordinated sequential activities. This involves establishing participation frameworks, awareness of conspecifics' attentional states, the monitoring of responses and moment-by-moment adjustment of signals to coordinate entry and exit phases of interactions, suspend and resume joint actions, request and respond to requests, and so forth, in contexts involving play, grooming, travelling or sharing food (e.g. [176,180,214,215]). Often, these finely coordinated interaction sequences involve a range of different visual articulators, including manual gestural actions, body orientation and gaze, and their detailed interplay has been illustrated nicely in these recent studies. Further advances may here be made by focusing on the subtle (and not so subtle) variation in the kinematic properties of these individual visual signals, as well as on the signals’ intricate temporal co-organization and potential differences in interactive meaning this may entail—an area that still requires considerable work in humans, too.

The above examples show that a focus beyond iconicity, including compositionality of multimodal utterances, movement kinematics and analyses of the role of visual signals for coordinating interactional processes, lays bare new potential precursors to human multimodal language and the dispositions that constitute the hypothesized interaction engine in modern humans [38,39]. To what extent exactly those aspects of visual behaviour appearing similar in humans reflect similarities in cognitive abilities, or their evolutionary roots, goes beyond the scope of the present review, and is to a large extent still a matter of future research. Detailed interaction analyses supplemented with experimental manipulation to causally relate behaviour to cognitive and social abilities may be the most promising way forward in trying to consolidate the meaning of observed parallels between human and non-human primates.

## 4. Human multimodal language and its potential precursors

While a range of theories about the origins of human language cast gesture as a central element, the weight and role attributed to the gestural modality varies considerably. Some theories ascribe a bridging function to gesture, serving as a transitional stage in the development of a fully-fledged vocal language system [3–8]. Others have cast the role of gesture as having emerged from an inherent biomechanical connection between vocalizations and bodily movements, suggesting that ‘hand gestural movements may thus have evolved as an embodied innovation for vocal control’ [210, p. 11366]. Both approaches can be considered speech-centric in that gesture fulfils functions that assist vocal language in achieving its full potential, be it via (proto-)symbolic representation or acoustic modulation.

The view put forward here contrasts with those approaches in that it considers visual signalling as being a fundamental component of modern human language. Thus, the present account is in line with existing ones arguing that human language evolved as a multimodal phenomenon (e.g. [9–18]) and that spoken language alone does not represent the evolutionary pinnacle and therefore, by itself, is not the explanandum. A multimodal communication system which evolved with the visual and the vocal channels inextricably intertwined (and on a par) is extremely effective as it offers deeply integrated but at the same time complementary modes of communication with different affordances for encoding information (linear-sequential versus holistic [34]).

Critically, such a multimodal, multilayered system allows for parallel information streams with rich, composite signals and multimodal utterances, which substantially expands expressive potential. This boosts the system's compositional power and affords complex but efficiently packaged—and by all likelihood more quickly recognizable—communicative *gestalts*. This rich expressive potential crucially rests on the diverse set of articulators humans have at their disposal (the hands, head, face, gaze and torso) and the wide range of visual signals they are able to convey and meaningfully combine. All this contributes to the versatile set of human pragmatic abilities. It also significantly augments the capacity for fine-tuning and flexibly adapting communicative messages to interlocutors and their interactive behaviours, a much-needed prerequisite for creating mutual understanding in social interaction.

**Figure 3.**
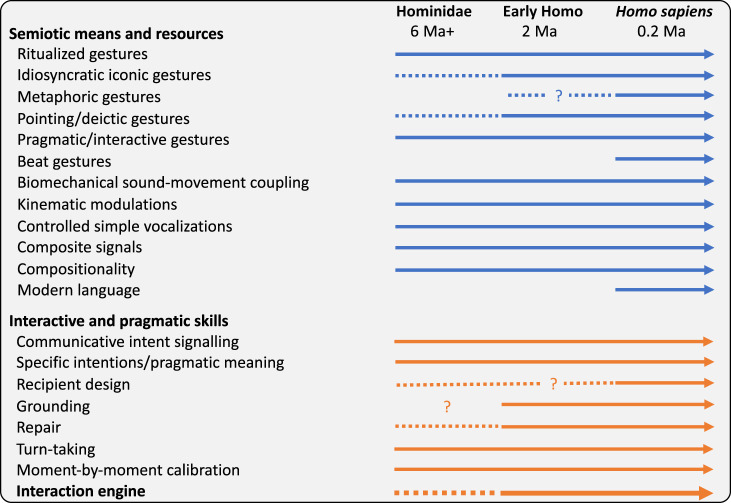
Hypothetical timelines for the evolution of hallmarks of human communicative behaviour. Solid arrows, present; dotted lines, precursor present; question marks, domain of uncertainty. Note that the gesture types refer to existing descriptions and labels (iconic, metaphoric, beats: McNeill [34]; pragmatic: Kendon [68]; interactive: Bavelas *et al*. [111]). Reference to the interaction engine is in bold since this hypothesized construct entails the interactive and pragmatic skills listed above it.

As Levinson [39] has pointed out, some non-human primate behaviours may be considered precursors to the human abilities that create a special capacity for social interaction, and thus the foundation for the emergence of modern human language. A multimodal view of human pragmatics and a consideration of visual bodily signals in the coordination of minds in interaction makes the gap between humans and non-human primate communication appear smaller than it may seem at first sight. As we saw above, such an approach points towards some of the key features of the modern human communication system already being present in our last common ancestor with chimpanzees around 6 Ma, or in even earlier last common ancestors on the hominid line (figure 3). Iconicity will of course have played a fundamental role in the further evolution of referential and propositional communication in both gesture and speech (e.g. [12,13,17,26,29,33]). The potential for early iconicity is already evident in non-human primates [29,165–167], plus perhaps even the seeds for some basic gesture–prosodic connection [216]. In line with the Interaction Engine Hypothesis, the main driver of creating the foundations for the emergence of complex language are likely to have been social-interactional abilities and motivations. Here I have highlighted that a focus on pragmatic, socially situated visual bodily signalling can reveal potentially even earlier evolutionary precursors to modern human language than commonly assumed.

The sections above have detailed a range of core contributions the body makes to communication. Delving deeper into investigating signals and their functions that scaffold the pragmatic processes elementary to coordinating minds in interaction is a fruitful way forward in our endeavour to understand both human and non-human primate communication in their own right. It also facilitates discovering potential precursors and the evolutionary path that led to the emergence of modern human multimodal language and its foundational interactional capacities.

## 5. Conclusion

Human communication is fundamentally multimodal. If we ask how verbal language evolved, then the answer has got to be not by supplanting gesture. Human visual signalling is anything but a temporary bridge to spoken language or a primitive mode of communication that was replaced by spoken words. Only a scenario in which the vocal and the visual modalities co-evolved into a tightly integrated system can explain how our species ended up with the highly adaptable and flexible multimodal toolbox that allows us to achieve the coordination of minds so effortlessly, effectively and efficiently. The multimodal nature of the human communication system combined with the socio-cognitive abilities that constitute the hypothesized interaction engine has equipped the human species with an unparalleled system for communicating not only semantic but also pragmatic meaning. A wide range of visual bodily signals going considerably beyond iconic manual gestures forms an integral component of the process of achieving mutual understanding in face-to-face interaction.

## Data Availability

This article has no additional data.

## Footnotes

1 Although this review is based on data and examples from speakers of different languages and cultures, these differences are glossed over in the present manuscript, as are individual differences and atypical populations. While all immensely important aspects in their own right, their discussion would go beyond the scope of the present review. Future analyses are required to devote detailed attention to these issues and gain an understanding of the extent to which current views may need to be refined.
